# Stress granules—membraneless organelles as therapeutic targets in pancreatic cancer

**DOI:** 10.1038/s44321-024-00040-2

**Published:** 2024-02-27

**Authors:** Carolin Schneider, Günter Schneider

**Affiliations:** 1 Department of General, Visceral and Pediatric Surgery, University Medical Center Göttingen, Göttingen, 37075 Germany; 2 Clinical Research Unit KFO5002, University Medical Center Goettingen, Goettingen, Germany; 3 CCC-N (Comprehensive Cancer Center Lower Saxony), Göttingen, 37075 Germany

**Keywords:** Cancer, Digestive System

## Abstract

G. Schneider & C. Schneider discuss the study by Santofimia-Castaño et al, in this issue of *EMBO Mol. Med.*, that shows that targeting NUPR1-dependent stress granules formation induces synthetic lethality in a mouse model of pancreatic cancer.

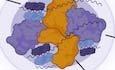

## Teaser

Therapies targeting pancreatic ductal adenocarcinoma (PDAC), driven in most cases by the KRAS oncogene, continue to present a clinical challenge. This emphasizes the need to explore alternative treatment paradigms in translational research. In this issue of *EMBO Mol. Med.*, Santofimia-Castano et al, highlight that stress-inducible nuclear protein 1 (NUPR1), an intrinsically disordered protein, serves as a regulator of KRAS-associated stress granules (SGs) (Santofimia-Castaño et al, 2024). Targeting NUPR1 by a small molecule inhibitor prevents SG formation as well as the development and progression of pancreatic intraepithelial neoplasia (PanINs). Moreover, the NUPR1 inhibitor ZW-115 triggers apoptosis of PDAC cells dependent on KRAS expression. In essence, these findings are supportive of targeting SGs as a therapeutic approach for PDAC (Santofimia-Castaño et al, 2024).

**Figure 1:**
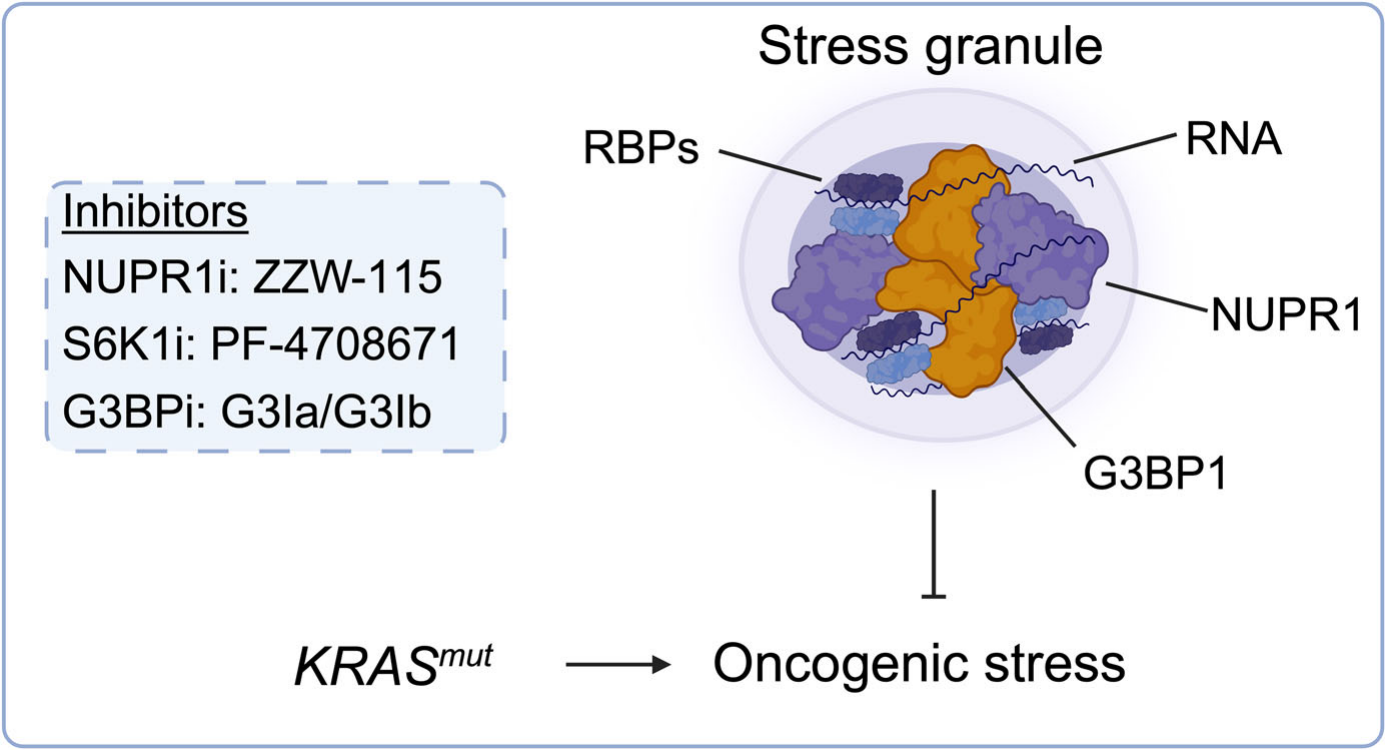
KRAS-associated stress granules (SGs). SGs, membraneless organelles (MLOs), protect PDAC from stress induced by mutant KRAS. Essential components of SGs, such as G3BP1, RNAs, RBPs, and the newly identified NUPR1 are illustrated. Small molecules targeting SGs were depicted. G3BP1: Ras GTPase-activating protein (GAP)-binding protein 1, NUPR1: Nuclear protein 1, RBP: RNA-binding protein, RNA: Ribonucleic acid.

The compartmentalization of processes and machineries into organelles is a fundamental concept in the biology of eukaryotic cells. Classic organelles, such as mitochondria or the endoplasmic reticulum, are enclosed by membranes. Additionally, membraneless organelles (MLOs), like the nucleolus, serve as functional units (Hirose et al, 2023). MLOs emerge as condensates formed by the multivalent interaction of nucleic acids and specific proteins through liquid-liquid phase separation (LLPS). The 0.1–2 μm sized stress granules (SGs) are cytoplasmatic MLOs induced by a variety of cellular stresses. While the precise role of SGs continues to be a subject of ongoing debate, the formation of SGs is often linked to stalled translation. Here, mRNAs, devoided of ribosomes, and RNA-binding proteins (RBPs), like Ras GTPase-activating protein (GAP)-binding protein 1(G3BP1) or T-cell-restricted intracellular antigen 1 (TIA1), undergo condensation. These SGs encompass a diverse array of hundreds of proteins and thousands of mRNAs. In cancers, SGs play a role as part of an adaptive response, enabling the cell to effectively cope with stressors, conferring a fitness advantage (Redding and Grabocka, 2023). The mutant *KRAS* oncogene, a central driver in PDAC, instigates the in vivo induction of SGs and primes for their formation under stress (Grabocka and Bar-Sagi, 2016) (Fig. 1). In obesity-associated PDAC, IGF1 plays a significant role in inducing signal transduction pathways that lead to the formation of SGs. Here, SGs are potential targets for inhibition via compounds disrupting the IGF1-induced PI3K signaling, like the S6K1 inhibitor PF-4708671 (Fonteneau et al, 2022) (Fig. 1). Furthermore, inhibiting SG formation in PDAC cells through G3BP1 knockdown diminishes cells’ resilience to oxidative stress in vitro and curtails tumor growth in vivo (Fonteneau et al, 2022). Compounds targeting G3BP1 are in development and are applicable to functionally investigate SGs (Freibaum et al, 2024) (Fig. 1). Together, such findings underscore the potential of targeting SGs as a strategy in combating PDAC, a concept extended by the study of Santofimia-Castano et al. The stress-inducible nuclear protein 1 (NUPR1) is an intrinsically disordered protein that is overexpressed in numerous tumor entities. Intrinsically disordered proteins are notably enriched in SGs, as they facilitate LLPS. By employing LC-MS/MS proteomics to delineate the NUPR1 interactome, the authors noted an enrichment of known SG-containing proteins under basal conditions, with a subsequent additional increase induced by metabolic or endoplasmic reticulum stress. The observation that NUPR1 interacts with G3BP1, a core component for SG condensation, and undergoes LLPS, indicates that the stress-inducible protein contributes to these MLOs. Moreover, comprehensive investigations involving both genetic loss-of-function studies and pharmacological inhibition experiments underscore the significance of NUPR1 in stress-induced SG formation, functionally expanding the core of SG regulators in the context of oncogenic KRAS. To assess the translational significance of targeting SGs containing NUPR1, the authors employed a NUPR1 inhibitor known as ZZW-115. This trifluoperazine-derived compound reduced SG formation in vitro and the efficacy of the compound was tightly connected to the expression of the KRAS oncogene. In addition, the authors used a well-characterized genetically engineered murine PDAC model, dependent on the pancreas-specific expression of KRAS^G12D^. Over time, KRAS^G12D^ induces the development of acinar-to-ductal metaplasia (ADM) and pancreatic intraepithelial neoplasia (PanIN), being a precursor of PDAC. Utilizing markers such as G3BP1, the authors observed SG formation in the murine model in vivo, consistent with former experimentation (Grabocka and Bar-Sagi, 2016). Remarkably, the administration of ZZW-115 to mice at an early time point, prior to the detectability of ADMs and PanINs, resulted in the complete prevention of lesion formation. The impact of ZZW-115 on PanIN progression was still noticeable when animals were treated at later time points, by which PanIN lesions had already developed. Mechanistically, the authors propose that NUPR1-inhibited cells are incapable of tolerating KRAS^G12D^-induced oncogenic stress, leading to the activation of caspase 3 and subsequent cell death. Hence, the administration of ZZW-115 mirrors the impaired progression of PanIN to PDAC observed in the absence of NUPR1.

In conclusion, the present study underscores the promising potential of SGs as viable targets in PDAC. Furthermore, it highlights a new pathway for intervention with KRAS^G12D^-associated SG formation, targetable by a small molecule NUPR1 inhibitor (Fig. 1). Further research is essential to deepen our comprehension of the role and function of SGs. This is particularly critical for customizing new therapies tailored to PDAC, given SGs’ documented role in constraining the effectiveness of targeted and chemotherapeutic treatments (Redding and Grabocka, 2023). Interestingly, a report from 2023 employed a proximity-dependent biotin identification (BioID) method utilizing G3BP1 and TIA1 as baits to profile SG proteins. In line with current understanding, the study detected enrichment of RNA- and ribosome-binding proteins. However, it also identified other classes of proteins, notably finding the sequestration of executioner caspase 3 and caspase 7 into SGs. The sequestration of executioner caspases into SGs has been observed to restrict apoptosis induction under specific experimental conditions in vitro. Furthermore, this mechanism could significantly impact tumor progression in vivo. For instance, preventing the sequestration of caspase 3 into stress granules has been shown to reduce tumor growth in a subcutaneous xenotransplant model of MKN28 cancer cells (Fujikawa et al, 2023). While the precise contribution of this mechanism to apoptosis upon SG targeting in the context of KRAS-mutated PDAC, as described by Santofimia-Castano et al, awaits further experimentation, these findings underscore the potential for functionalizing stress granule content to comprehend their role in tumor progression and therapy adaptation. It is important to acknowledge that the assembly and disassembly of SGs are controlled and dynamic processes, assuring that the content of SGs adapts to meet cellular demands. For instance, the N7-methylguanosine (m7G) modification, internally installed in mRNAs by the methyltransferase complex METTL1-WDR4, is recognized by Quaking (QKIs) RNA-binding proteins. QKI7 facilitates the trafficking of internally m7G-modified mRNAs to SGs, thereby modulating sensitivity to doxorubicin (Zhao et al, 2023), which demonstrates the relevance of controlled trafficking of mRNAs to SGs. Furthermore, research into proteome trafficking between the nucleus and SGs has revealed that stress induced by arsenite triggers a transient localization of the AP1 transcription factor JUN to stress granules. JUN’s localization to SGs serves as a protective mechanism against aggregation and degradation due to oxidative stress. This safeguarded state in SGs ensures that JUN remains available to rejuvenate AP1 activity during the stress recovery phase (Qin et al, 2023). It remains to be explored whether SGs function as protective reservoirs for proteins, enabling rapid adaptation to the fluctuating demands of the hostile tumor microenvironment of KRAS-driven PDAC.
